# Supplementary material for: Is the future already here? The impact of climate change on the distribution of the eastern coral snake (Micrurus fulvius)
Source: PeerJ. 2018 May 1;6:e4647. doi: 10.7717/peerj.4647 (PMC5935076; doi:10.7717/peerj.4647)
Supplement: Supplemental Information 1 [file peerj-06-4647-s001.docx]

Metadata

The two raw data files include filtered locality points that were used in the final model.

“Locality_pts_1950_2000” includes the following columns:

Species – all Micrurus fulvius

Longitude – in WGS84 geographic projection

Latitude – in WGS84 geographic projection

Uncertainty in km – estimated as explained in methods

Year Collected – all between 1950 and 2000

Data Source –

- ASU: Arizona State University
- gbif: [www.gbif.org](http://www.gbif.org)
- HerpNet: from [www.herpnet.org](http://www.herpnet.org), which has since been folded into [www.vertnet.org](http://www.vertnet.org)
- idigbio: www.idigbio.org
- MISS: Mississippi Museum of Natural History
- NCSM: NC Museum of Natural Sciences
- UCH originated at the Charleston Museum and are now part of the NC Museum of Natural Sciences

Catalog Number – a number used by the specific museum or collection to identify the vouchered specimen
