# Supplementary material for: Is the future already here? The impact of climate change on the distribution of the eastern coral snake (Micrurus fulvius)
Source: PeerJ. 2018 May 1;6:e4647. doi: 10.7717/peerj.4647 (PMC5935076; doi:10.7717/peerj.4647)
Supplement: Appendix S1 [file peerj-06-4647-s004.docx]

Appendix S1. Museums that contributed locality data for coral snakes, either directly via the curator, or through the gbif ([www.gbif.org](http://www.gbif.org)), vertnet ([www.vertnet.org](http://www.vertnet.org)), or idigbio (www.idigbio.org) databases.

| Inst. Code | Institution Name |
| --- | --- |
| ANSP | The Academy of Natural Sciences, Philadelphia, PA |
| AUM | Auburn University Natural History Museum and Learning Center |
| CAS | California Academy of Sciences, San Francisco, CA |
| CM | Carnegie Museum of Natural History, Philadelphia, PA |
| CU | Cornell University Museum of Vertebrates |
| FLMNH | Florida Museum of Natural History, Gainesville, FL |
| FMNH | Field Museum of Natural History, Chicago, IL |
| GMNH | Georgia Museum of Natural History, University of Georgia |
| GSU | Georgia State University |
| INHS | Illinois Natural History Survey, University of Illinois |
| KU | University of Kansas, Lawrence, KS |
| LACM | Natural History Museum of Los Angeles County |
| LSU | Louisiana Museum of Natural History, Louisiana State University |
| MCZ | Museum of Comparative Zoology, Harvard University |
| MHP | Museum of the High Plains - Fort Hays State University, Sternberg Museum of Natural History, Fort Hays, KS |
| MISS | Mississippi Museum of Natural History |
| MPM | Milwaukee Public Museum |
| MSB | Museum of Southwestern Biology, University of New Mexico |
| MSU | Division of Vertebrate Natural History, Michigan State University Museum |
| MSUM | Minnesota State University Moorhead |
| MVZ | Museum of Vertebrate Zoology, University of California, Berkeley |
| NCSM | North Carolina State Museum of Natural Sciences, now the North Carolina Museum of Natural Sciences |
| NLU | University of Louisiana at Monroe |
| OMNH | Sam Noble Oklahoma Museum, University of Oklahoma |
| PSM | James R. Slater Museum, University of Puget Sound |
| ROM | Royal Ontario Museum |
| SDNHM | San Diego Natural History Museum |
| TCWC | Texas Cooperative Wildlife Collection, Texas A & M University |
| UA | University of Alabama Museum of Natural History |
| UCM | University of Colorado Museum |
| UIMNH | University of Iowa Museum of Natural History |
| USNM | Smithsonian National Museum of Natural History, Washington, D.C. |
| UTEP | The Centennial Museum, University of Texas at El Paso |
| YPM | Peabody Museum, Yale University |
| ZMUC | Zoological Museum, University of Copenhagen |
