# Supplementary material for: Is the future already here? The impact of climate change on the distribution of the eastern coral snake (Micrurus fulvius)
Source: PeerJ. 2018 May 1;6:e4647. doi: 10.7717/peerj.4647 (PMC5935076; doi:10.7717/peerj.4647)
Supplement: Appendix S2 — Bioclimatic variables came from WorldClim ( http://www.worldclim.org/) and soil type from the Harmonized World Soil Database ( http://www.arcgis.com/home/item.html?id=1d16ed2a0aa24ab39e5ee6c491965883). Temperature is expressed in degrees Celsius multiplied by 10, precipitation is measured in mm, and soil type is a categorical variable with specific categories listed below. [file peerj-06-4647-s005.zip › Appendix S2.docx]

Appendix S2. All environmental layers used in the current climate modeling shown for the study area. Bioclimatic variables came from WorldClim (<http://www.worldclim.org/>) and soil type from the Harmonized World Soil Database (http://www.arcgis.com/home/item.html?id=1d16ed2a0aa24ab39e5ee6c491965883 ). Temperature is expressed in degrees Celsius multiplied by 10, precipitation is measured in mm, and soil type is a categorical variable with specific categories listed below.

Bio1 = Annual Mean Temperature
Bio2 = Mean Diurnal Range (Mean of monthly (max temp - min temp))
Bio3 = Isothermality (BIO2/BIO7) (* 100)
Bio4 = Temperature Seasonality (standard deviation *100)
Bio5 = Max Temperature of Warmest Month
Bio6 = Min Temperature of Coldest Month
Bio7 = Temperature Annual Range (BIO5-BIO6)
Bio8 = Mean Temperature of Wettest Quarter
Bio9 = Mean Temperature of Driest Quarter
Bio10 = Mean Temperature of Warmest Quarter
Bio11 = Mean Temperature of Coldest Quarter
Bio12 = Annual Precipitation
Bio13 = Precipitation of Wettest Month
Bio14 = Precipitation of Driest Month
Bio15 = Precipitation Seasonality (Coefficient of Variation)
Bio16 = Precipitation of Wettest Quarter
Bio17 = Precipitation of Driest Quarter
Bio18 = Precipitation of Warmest Quarter
Bio19 = Precipitation of Coldest Quarter

Soil = Soil Type, codes are listed below:

AC = Soils with subsurface accumulation of low activity clays and low base saturation

AR = Sandy soils featuring very weak or no soil development

CL = Soils with accumulation of secondary calcium carbonates

CM = Weakly to moderately developed soils

GL = Soils with permanent or temporary wetness near the surface

HS = Soils which are composed of organic materials

LV = Soils with subsurface accumulation of high activity clays and high base saturation

LX = Soils with subsurface accumulation of low activity clays and high base saturation

PT = Wet soils with an irreversibly hardening mixture of iron, clay and quartz in the

Subsoil

PZ = Acid soils with a subsurface accumulation of iron-aluminum-organic

VR = Dark-colored cracking and swelling clays

WR = Water Bodies
