# Supplementary figures and images for: Is the future already here? The impact of climate change on the distribution of the eastern coral snake (Micrurus fulvius)
Source: PeerJ. 2018 May 1;6:e4647. doi: 10.7717/peerj.4647 (PMC5935076; doi:10.7717/peerj.4647)

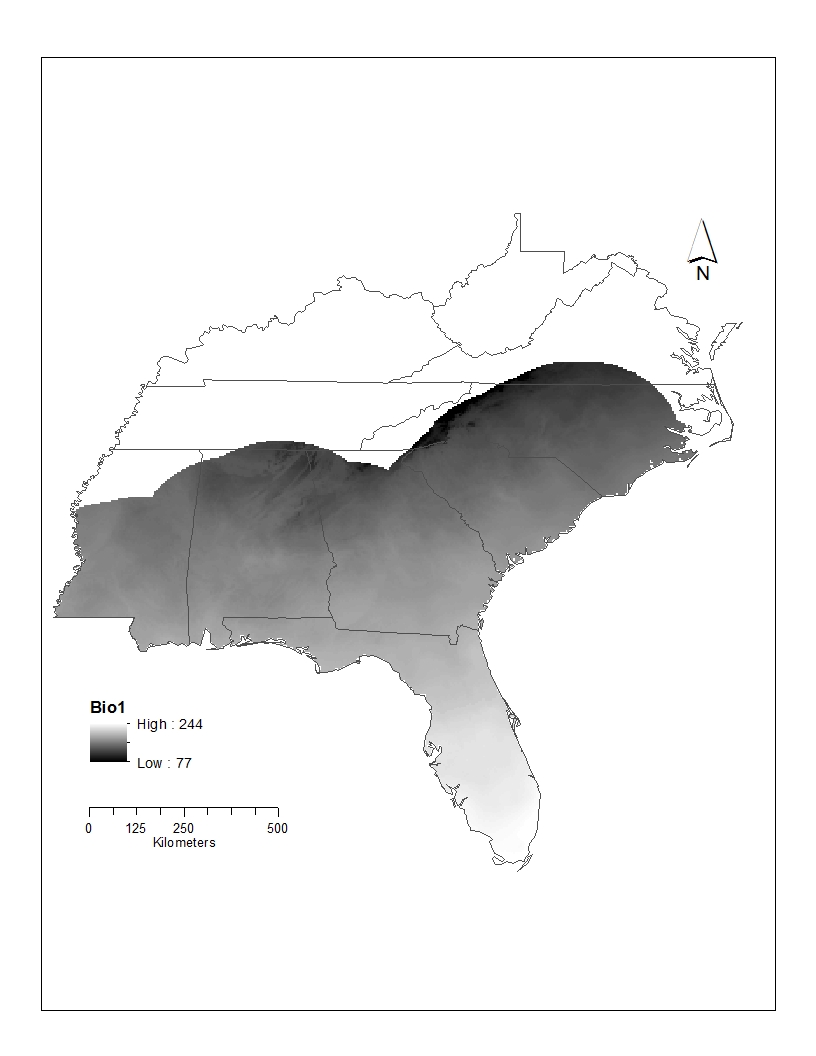

Supplement: Appendix S2 — Bioclimatic variables came from WorldClim ( http://www.worldclim.org/) and soil type from the Harmonized World Soil Database ( http://www.arcgis.com/home/item.html?id=1d16ed2a0aa24ab39e5ee6c491965883). Temperature is expressed in degrees Celsius multiplied by 10, precipitation is measured in mm, and soil type is a categorical variable with specific categories listed below. [file peerj-06-4647-s005.zip › Bio1.jpg]

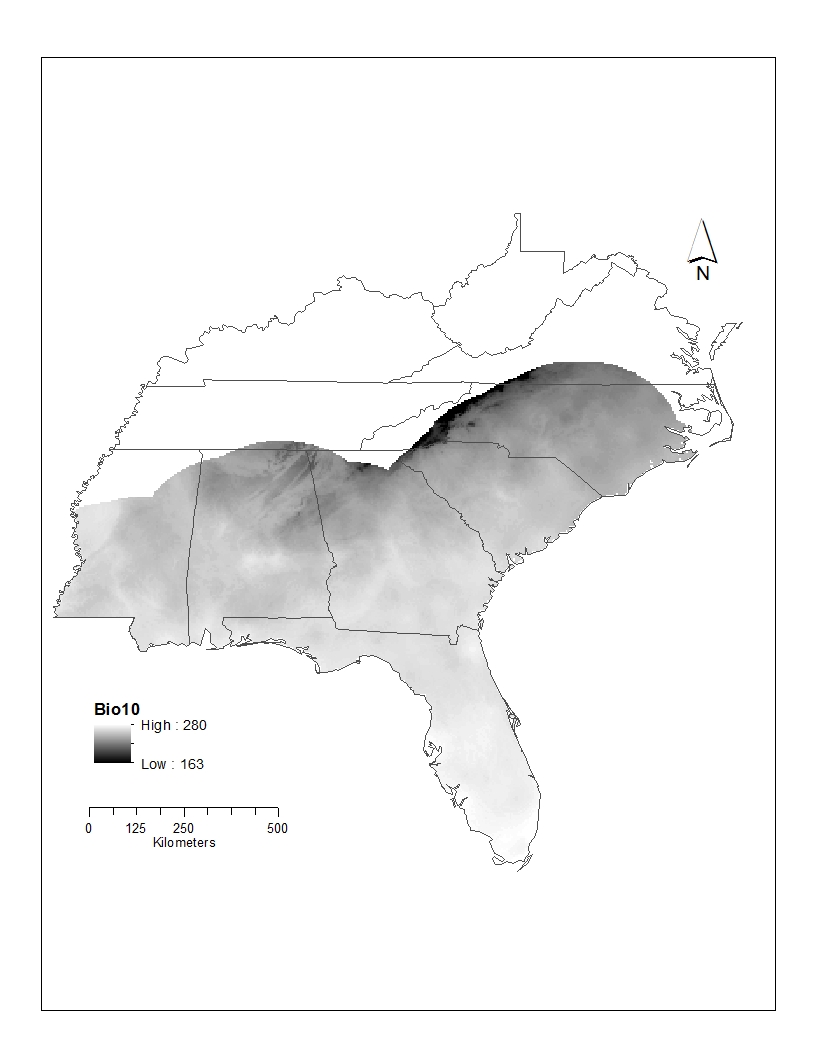

Supplement: Appendix S2 — Bioclimatic variables came from WorldClim ( http://www.worldclim.org/) and soil type from the Harmonized World Soil Database ( http://www.arcgis.com/home/item.html?id=1d16ed2a0aa24ab39e5ee6c491965883). Temperature is expressed in degrees Celsius multiplied by 10, precipitation is measured in mm, and soil type is a categorical variable with specific categories listed below. [file peerj-06-4647-s005.zip › Bio10.jpg]

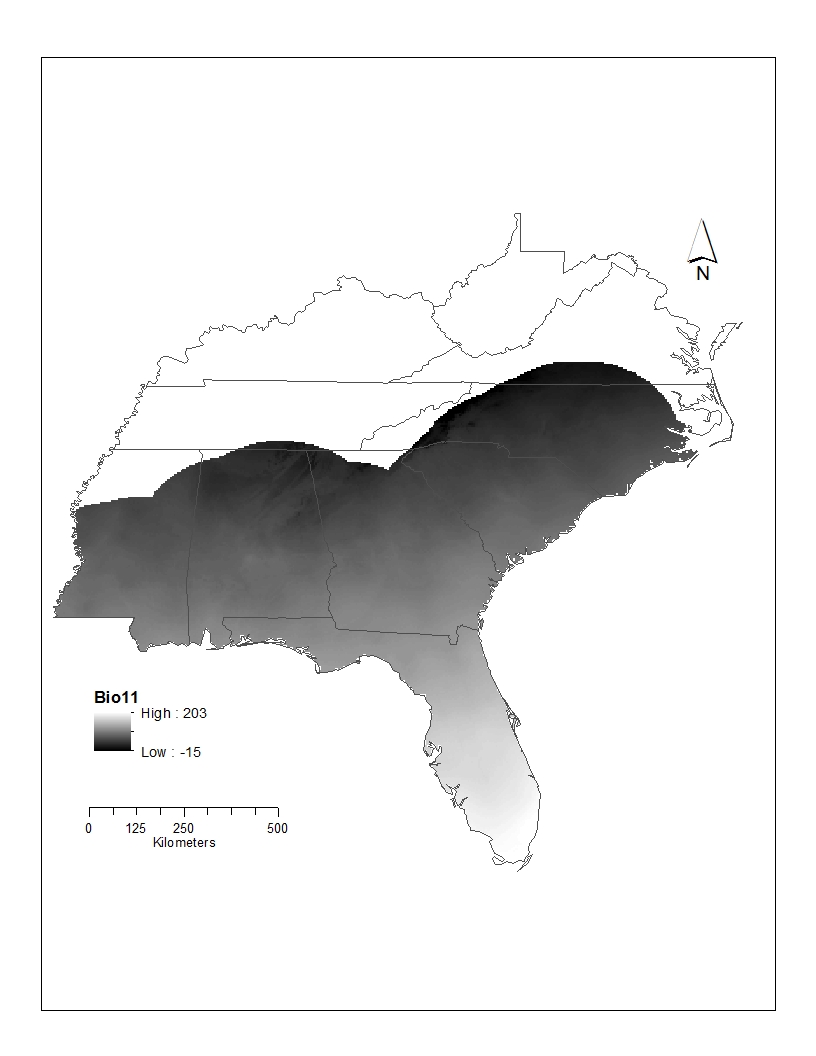

Supplement: Appendix S2 — Bioclimatic variables came from WorldClim ( http://www.worldclim.org/) and soil type from the Harmonized World Soil Database ( http://www.arcgis.com/home/item.html?id=1d16ed2a0aa24ab39e5ee6c491965883). Temperature is expressed in degrees Celsius multiplied by 10, precipitation is measured in mm, and soil type is a categorical variable with specific categories listed below. [file peerj-06-4647-s005.zip › Bio11.jpg]

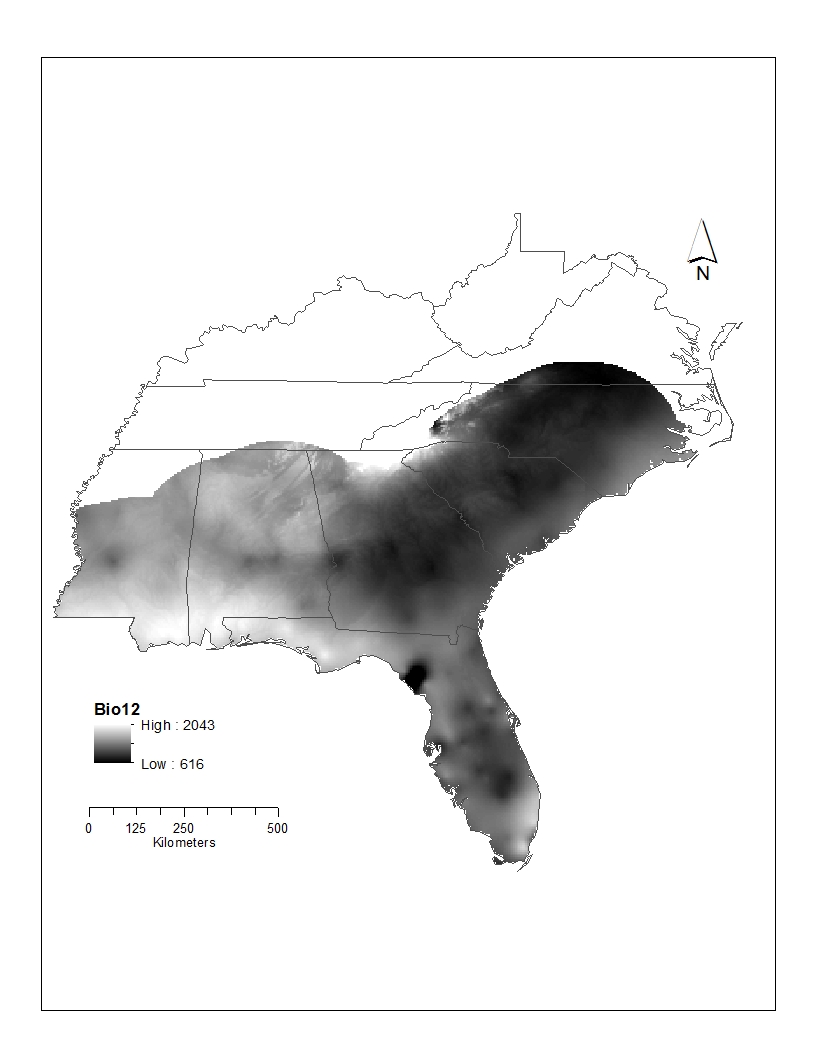

Supplement: Appendix S2 — Bioclimatic variables came from WorldClim ( http://www.worldclim.org/) and soil type from the Harmonized World Soil Database ( http://www.arcgis.com/home/item.html?id=1d16ed2a0aa24ab39e5ee6c491965883). Temperature is expressed in degrees Celsius multiplied by 10, precipitation is measured in mm, and soil type is a categorical variable with specific categories listed below. [file peerj-06-4647-s005.zip › Bio12.jpg]

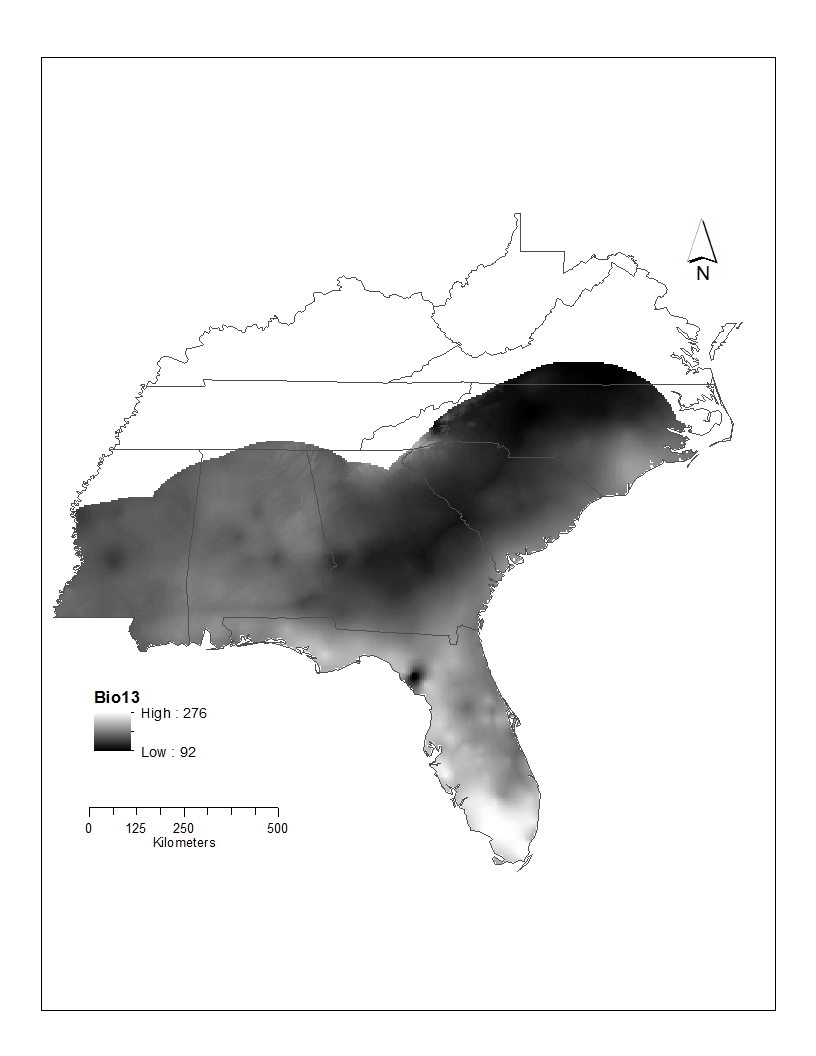

Supplement: Appendix S2 — Bioclimatic variables came from WorldClim ( http://www.worldclim.org/) and soil type from the Harmonized World Soil Database ( http://www.arcgis.com/home/item.html?id=1d16ed2a0aa24ab39e5ee6c491965883). Temperature is expressed in degrees Celsius multiplied by 10, precipitation is measured in mm, and soil type is a categorical variable with specific categories listed below. [file peerj-06-4647-s005.zip › Bio13.jpg]

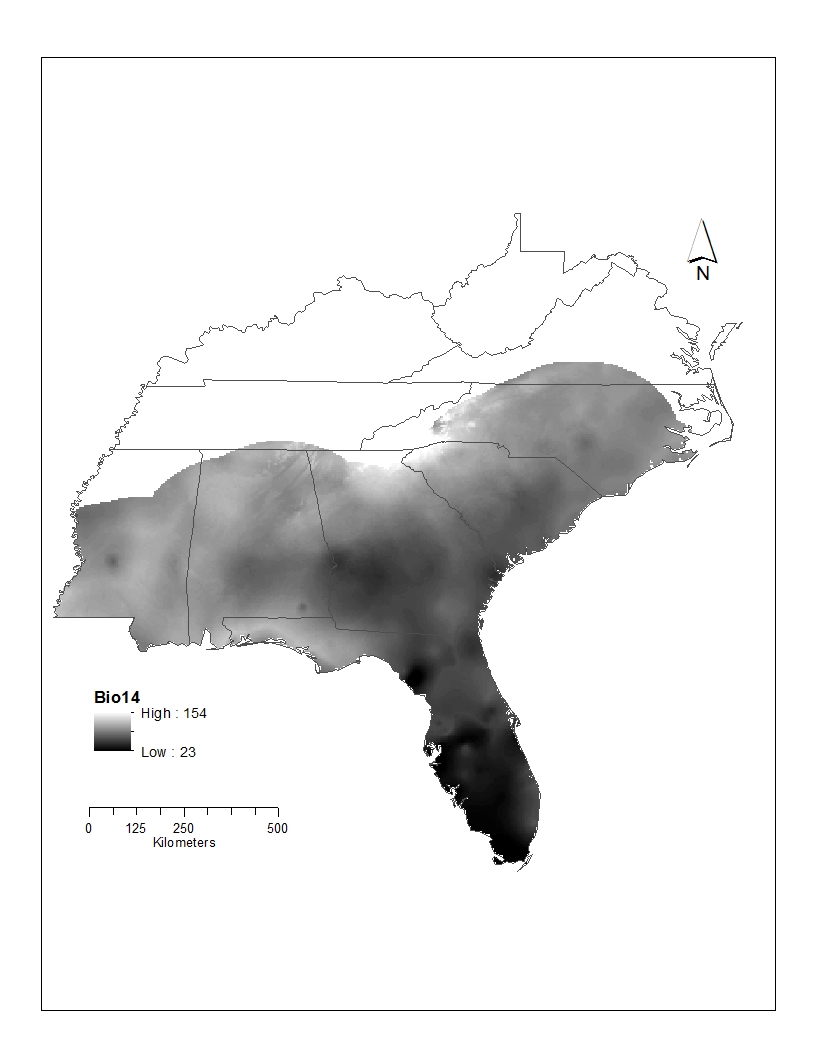

Supplement: Appendix S2 — Bioclimatic variables came from WorldClim ( http://www.worldclim.org/) and soil type from the Harmonized World Soil Database ( http://www.arcgis.com/home/item.html?id=1d16ed2a0aa24ab39e5ee6c491965883). Temperature is expressed in degrees Celsius multiplied by 10, precipitation is measured in mm, and soil type is a categorical variable with specific categories listed below. [file peerj-06-4647-s005.zip › Bio14.jpg]

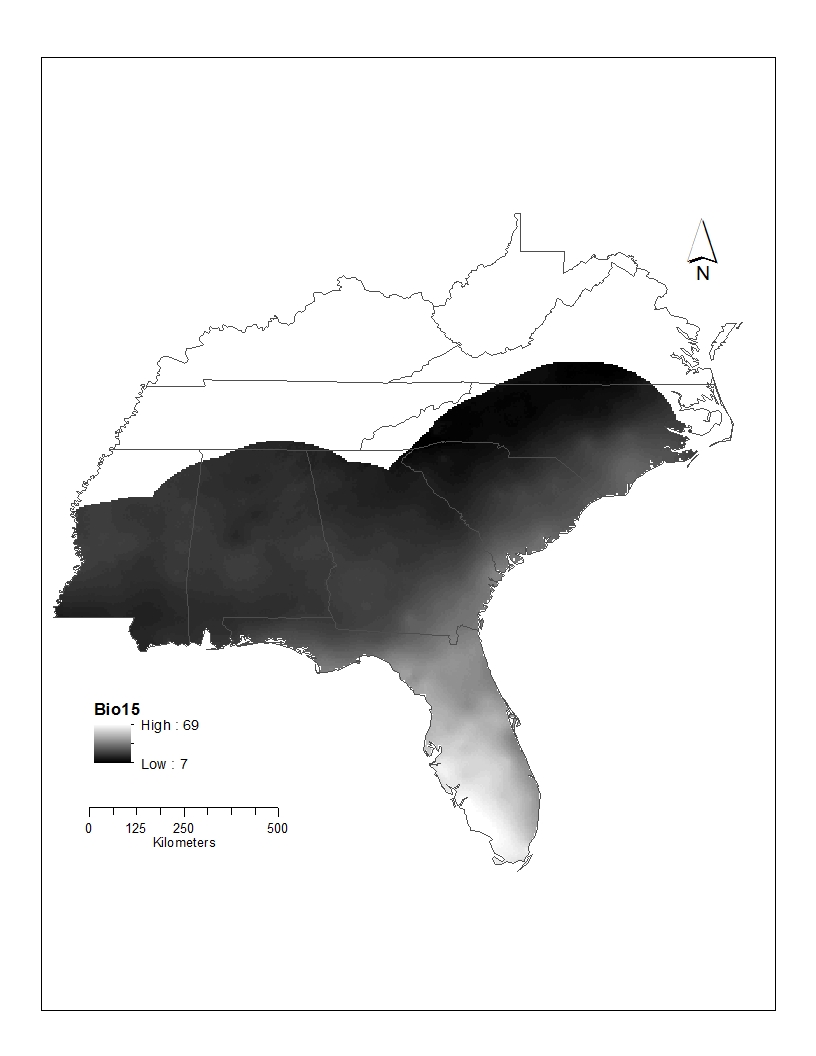

Supplement: Appendix S2 — Bioclimatic variables came from WorldClim ( http://www.worldclim.org/) and soil type from the Harmonized World Soil Database ( http://www.arcgis.com/home/item.html?id=1d16ed2a0aa24ab39e5ee6c491965883). Temperature is expressed in degrees Celsius multiplied by 10, precipitation is measured in mm, and soil type is a categorical variable with specific categories listed below. [file peerj-06-4647-s005.zip › Bio15.jpg]

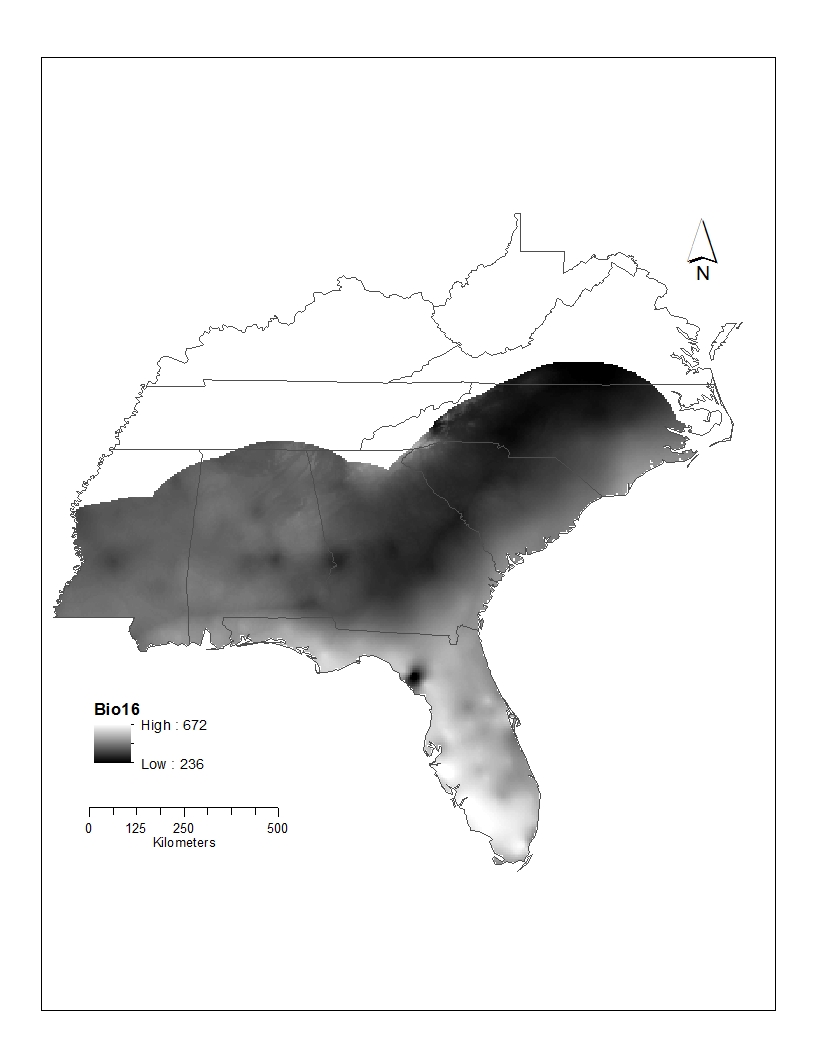

Supplement: Appendix S2 — Bioclimatic variables came from WorldClim ( http://www.worldclim.org/) and soil type from the Harmonized World Soil Database ( http://www.arcgis.com/home/item.html?id=1d16ed2a0aa24ab39e5ee6c491965883). Temperature is expressed in degrees Celsius multiplied by 10, precipitation is measured in mm, and soil type is a categorical variable with specific categories listed below. [file peerj-06-4647-s005.zip › Bio16.jpg]

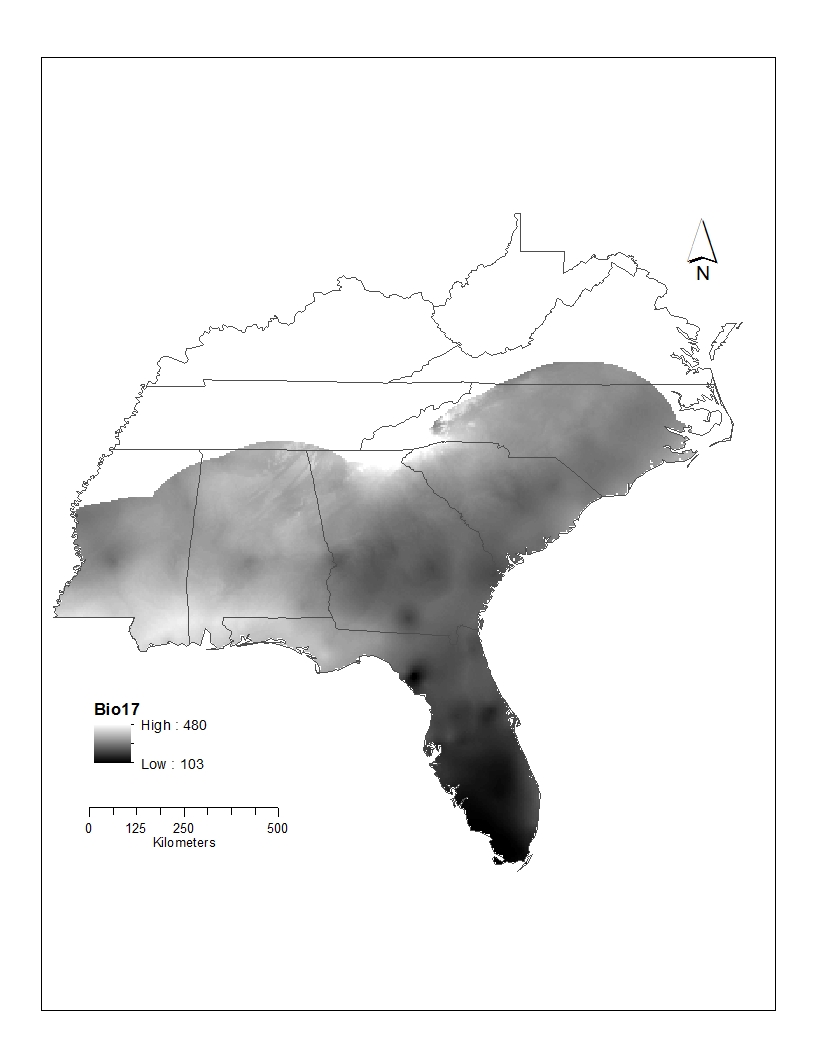

Supplement: Appendix S2 — Bioclimatic variables came from WorldClim ( http://www.worldclim.org/) and soil type from the Harmonized World Soil Database ( http://www.arcgis.com/home/item.html?id=1d16ed2a0aa24ab39e5ee6c491965883). Temperature is expressed in degrees Celsius multiplied by 10, precipitation is measured in mm, and soil type is a categorical variable with specific categories listed below. [file peerj-06-4647-s005.zip › Bio17.jpg]

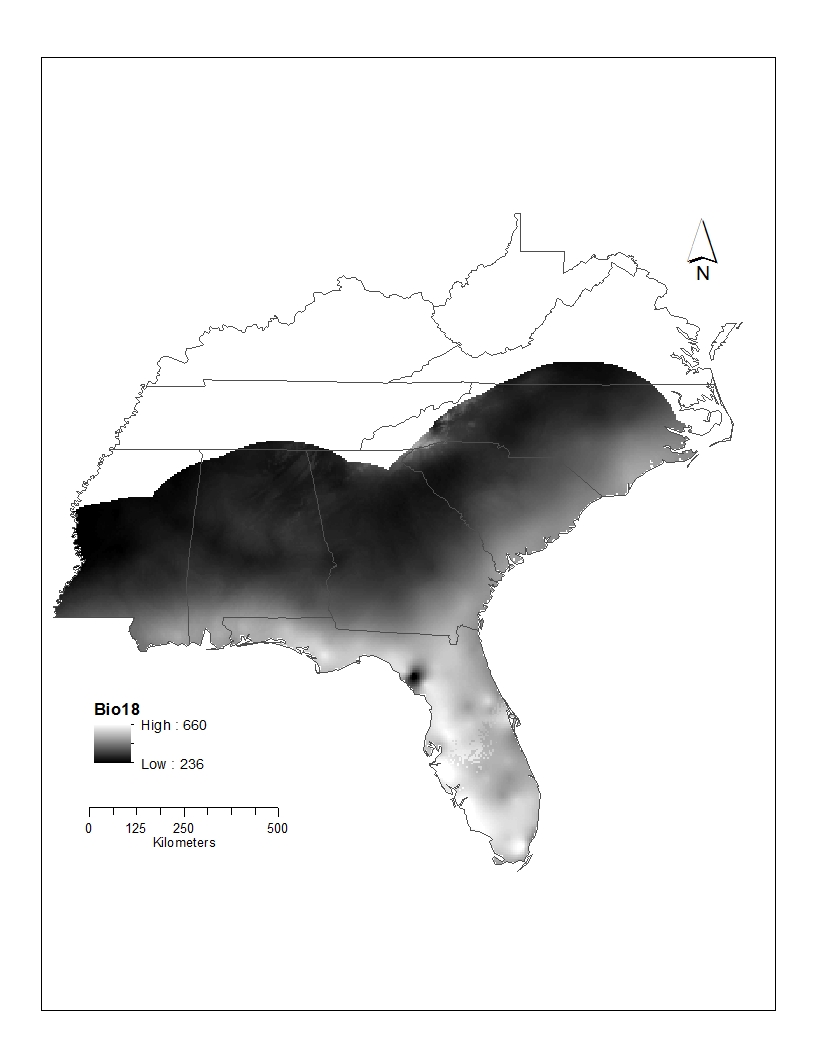

Supplement: Appendix S2 — Bioclimatic variables came from WorldClim ( http://www.worldclim.org/) and soil type from the Harmonized World Soil Database ( http://www.arcgis.com/home/item.html?id=1d16ed2a0aa24ab39e5ee6c491965883). Temperature is expressed in degrees Celsius multiplied by 10, precipitation is measured in mm, and soil type is a categorical variable with specific categories listed below. [file peerj-06-4647-s005.zip › Bio18.jpg]

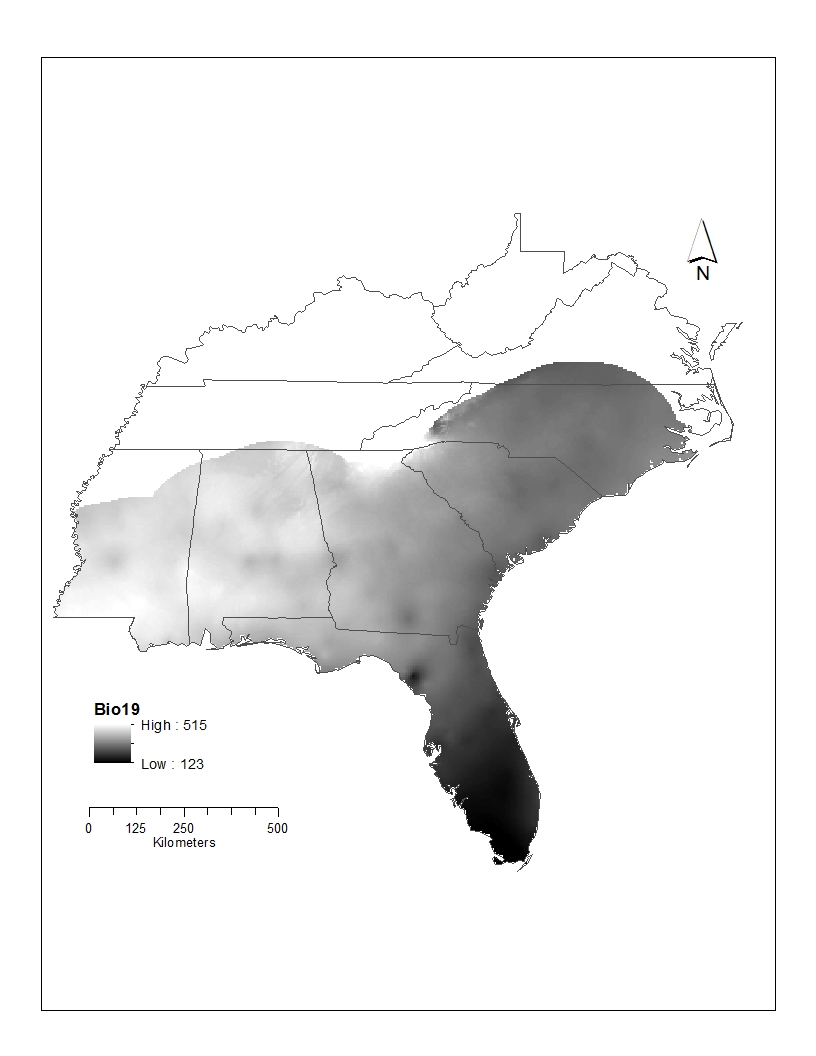

Supplement: Appendix S2 — Bioclimatic variables came from WorldClim ( http://www.worldclim.org/) and soil type from the Harmonized World Soil Database ( http://www.arcgis.com/home/item.html?id=1d16ed2a0aa24ab39e5ee6c491965883). Temperature is expressed in degrees Celsius multiplied by 10, precipitation is measured in mm, and soil type is a categorical variable with specific categories listed below. [file peerj-06-4647-s005.zip › Bio19.jpg]

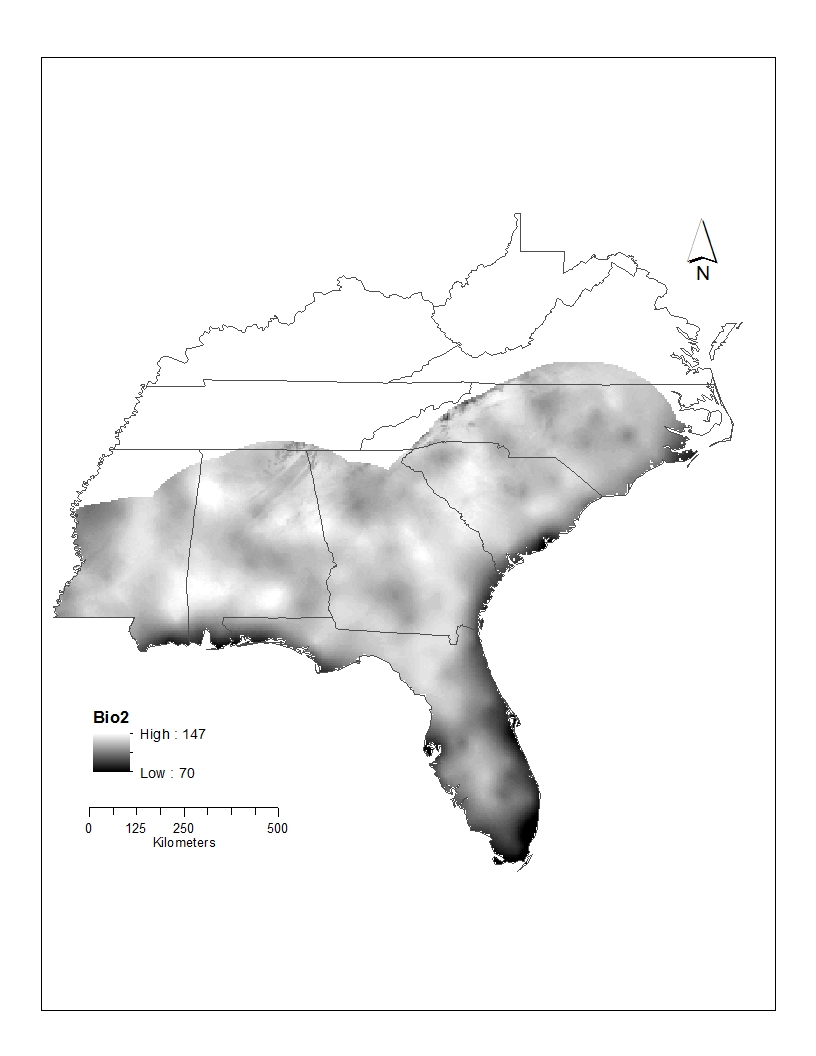

Supplement: Appendix S2 — Bioclimatic variables came from WorldClim ( http://www.worldclim.org/) and soil type from the Harmonized World Soil Database ( http://www.arcgis.com/home/item.html?id=1d16ed2a0aa24ab39e5ee6c491965883). Temperature is expressed in degrees Celsius multiplied by 10, precipitation is measured in mm, and soil type is a categorical variable with specific categories listed below. [file peerj-06-4647-s005.zip › Bio2.jpg]

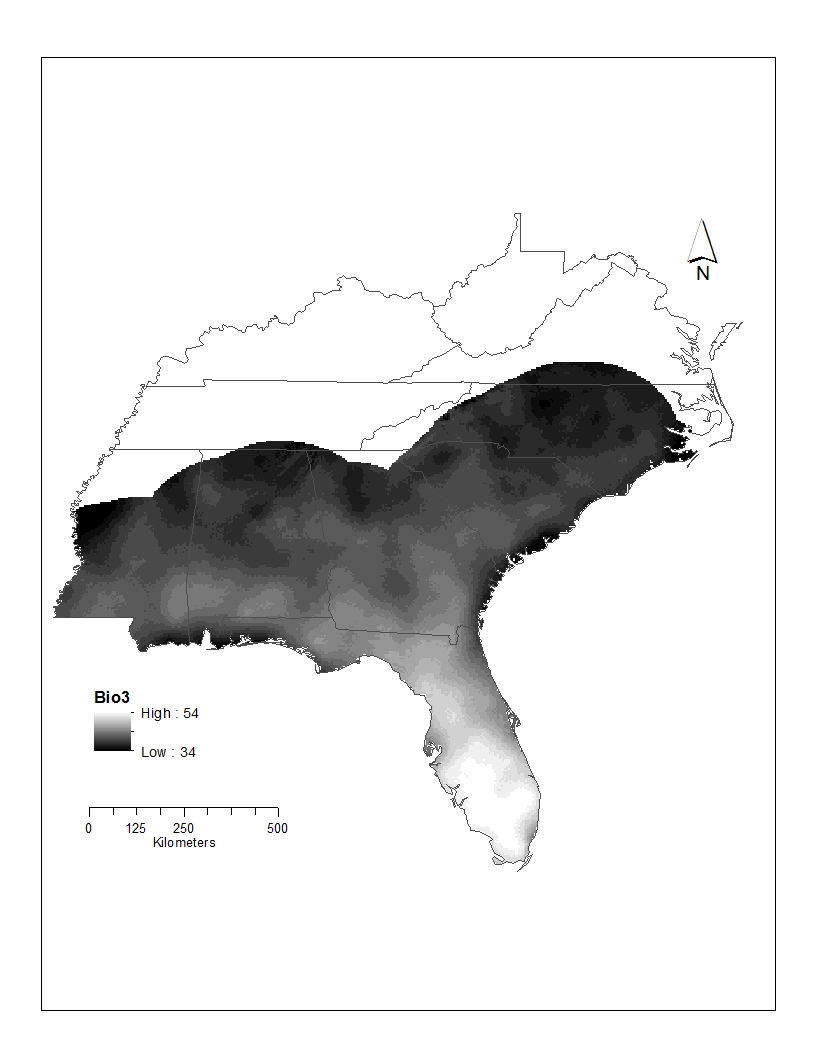

Supplement: Appendix S2 — Bioclimatic variables came from WorldClim ( http://www.worldclim.org/) and soil type from the Harmonized World Soil Database ( http://www.arcgis.com/home/item.html?id=1d16ed2a0aa24ab39e5ee6c491965883). Temperature is expressed in degrees Celsius multiplied by 10, precipitation is measured in mm, and soil type is a categorical variable with specific categories listed below. [file peerj-06-4647-s005.zip › Bio3.jpg]

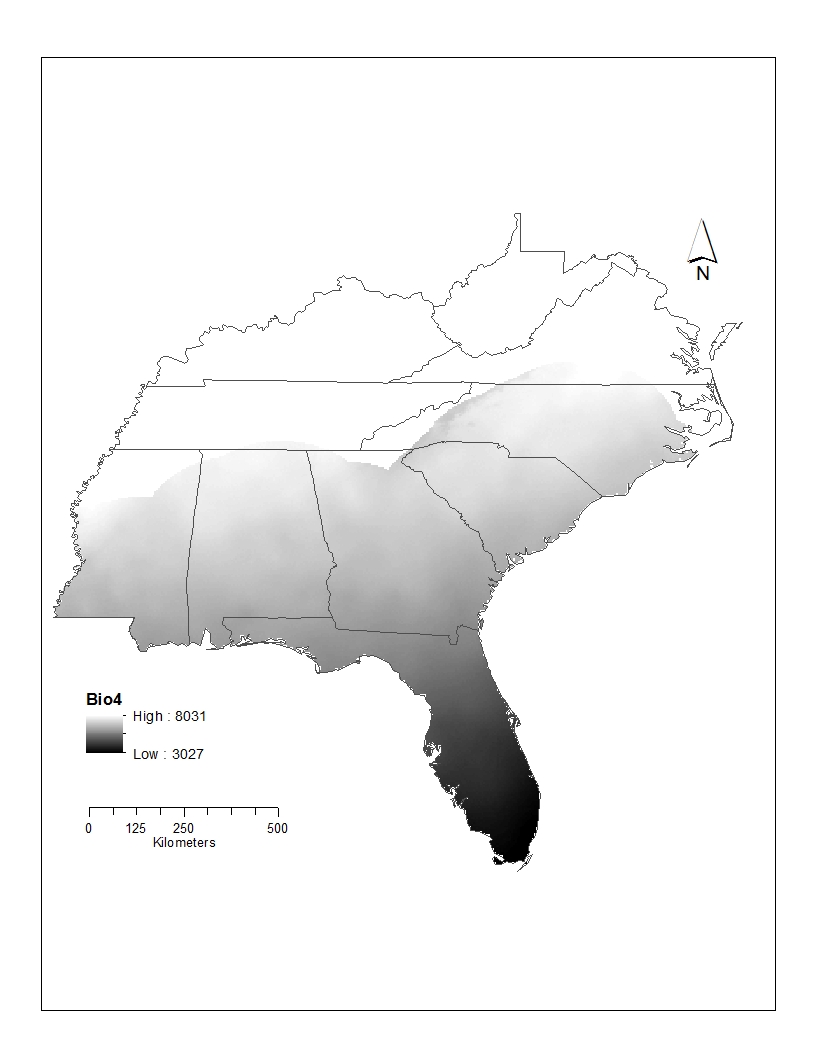

Supplement: Appendix S2 — Bioclimatic variables came from WorldClim ( http://www.worldclim.org/) and soil type from the Harmonized World Soil Database ( http://www.arcgis.com/home/item.html?id=1d16ed2a0aa24ab39e5ee6c491965883). Temperature is expressed in degrees Celsius multiplied by 10, precipitation is measured in mm, and soil type is a categorical variable with specific categories listed below. [file peerj-06-4647-s005.zip › Bio4.jpg]

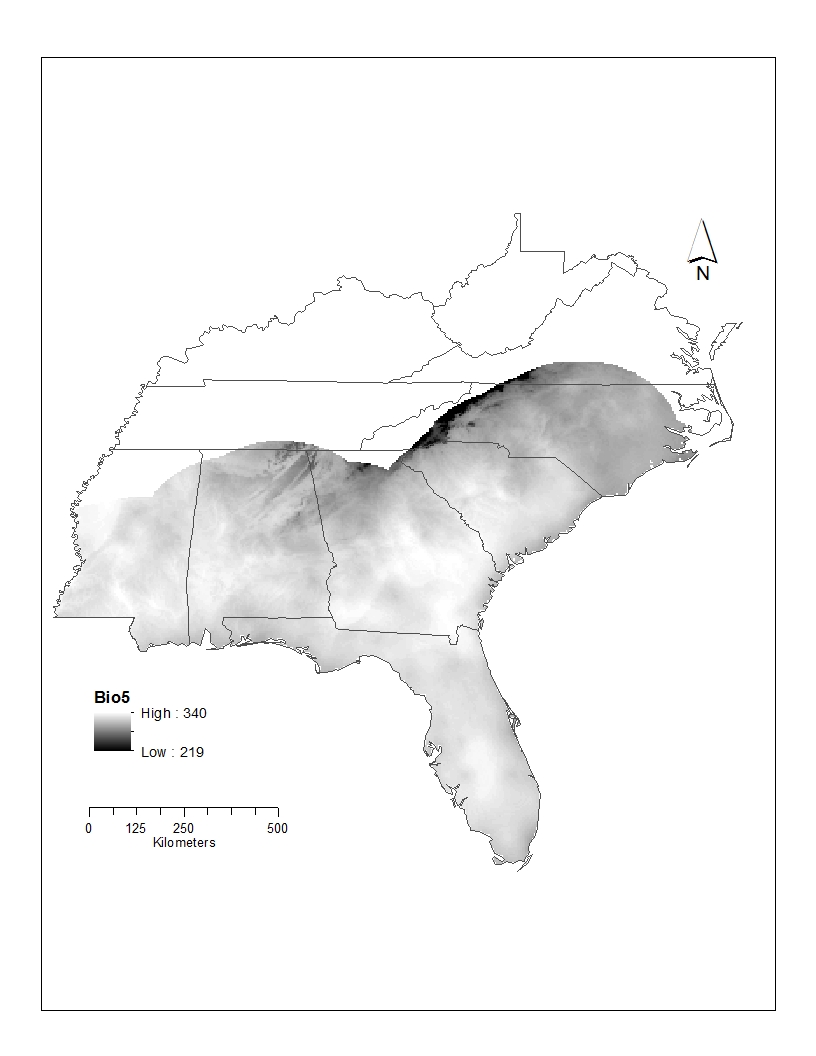

Supplement: Appendix S2 — Bioclimatic variables came from WorldClim ( http://www.worldclim.org/) and soil type from the Harmonized World Soil Database ( http://www.arcgis.com/home/item.html?id=1d16ed2a0aa24ab39e5ee6c491965883). Temperature is expressed in degrees Celsius multiplied by 10, precipitation is measured in mm, and soil type is a categorical variable with specific categories listed below. [file peerj-06-4647-s005.zip › Bio5.jpg]

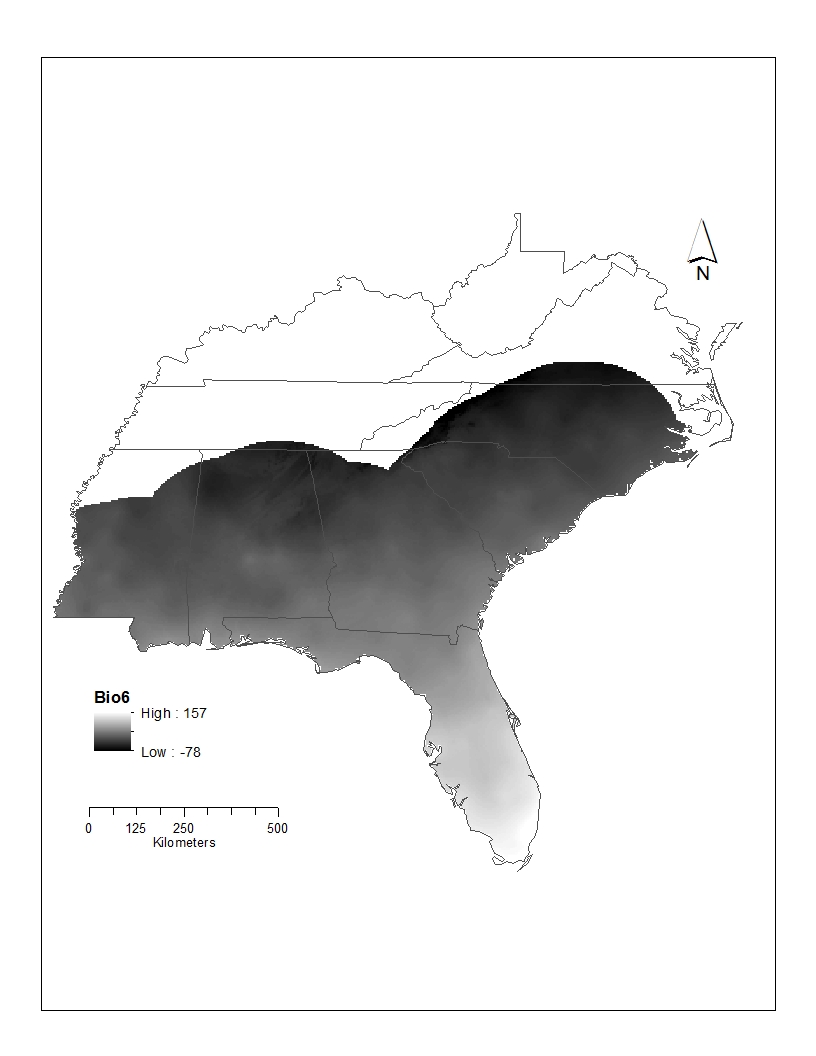

Supplement: Appendix S2 — Bioclimatic variables came from WorldClim ( http://www.worldclim.org/) and soil type from the Harmonized World Soil Database ( http://www.arcgis.com/home/item.html?id=1d16ed2a0aa24ab39e5ee6c491965883). Temperature is expressed in degrees Celsius multiplied by 10, precipitation is measured in mm, and soil type is a categorical variable with specific categories listed below. [file peerj-06-4647-s005.zip › Bio6.jpg]

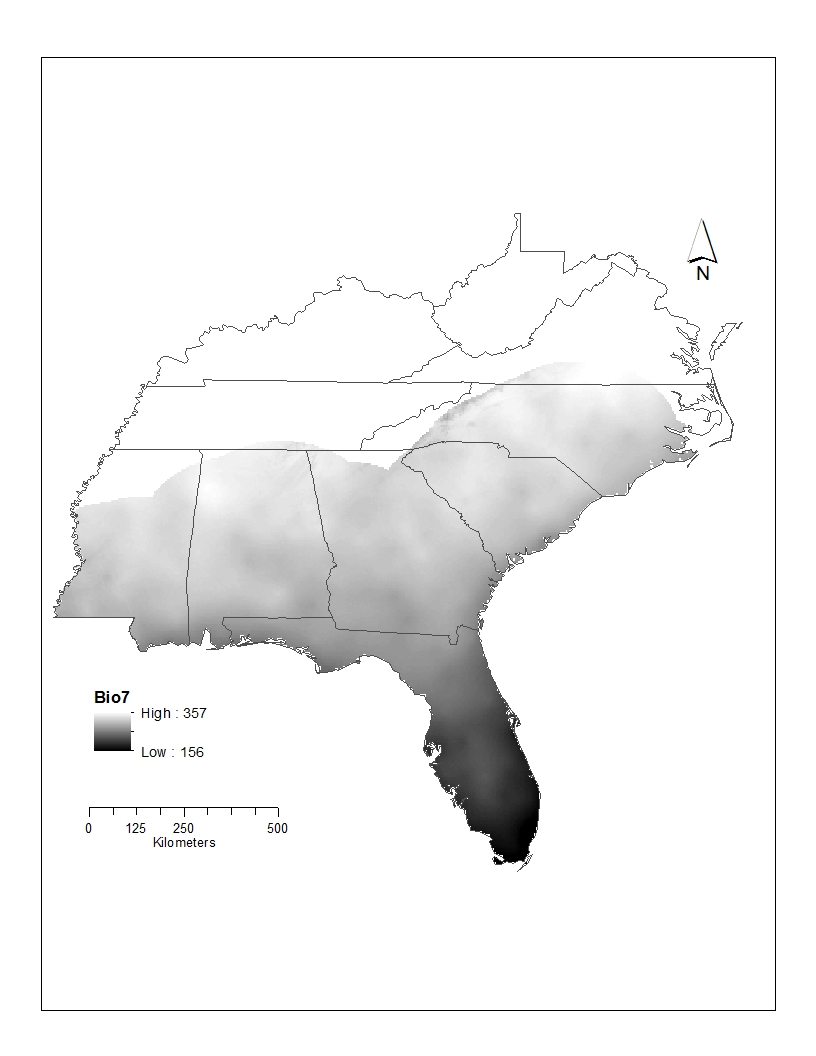

Supplement: Appendix S2 — Bioclimatic variables came from WorldClim ( http://www.worldclim.org/) and soil type from the Harmonized World Soil Database ( http://www.arcgis.com/home/item.html?id=1d16ed2a0aa24ab39e5ee6c491965883). Temperature is expressed in degrees Celsius multiplied by 10, precipitation is measured in mm, and soil type is a categorical variable with specific categories listed below. [file peerj-06-4647-s005.zip › Bio7.jpg]

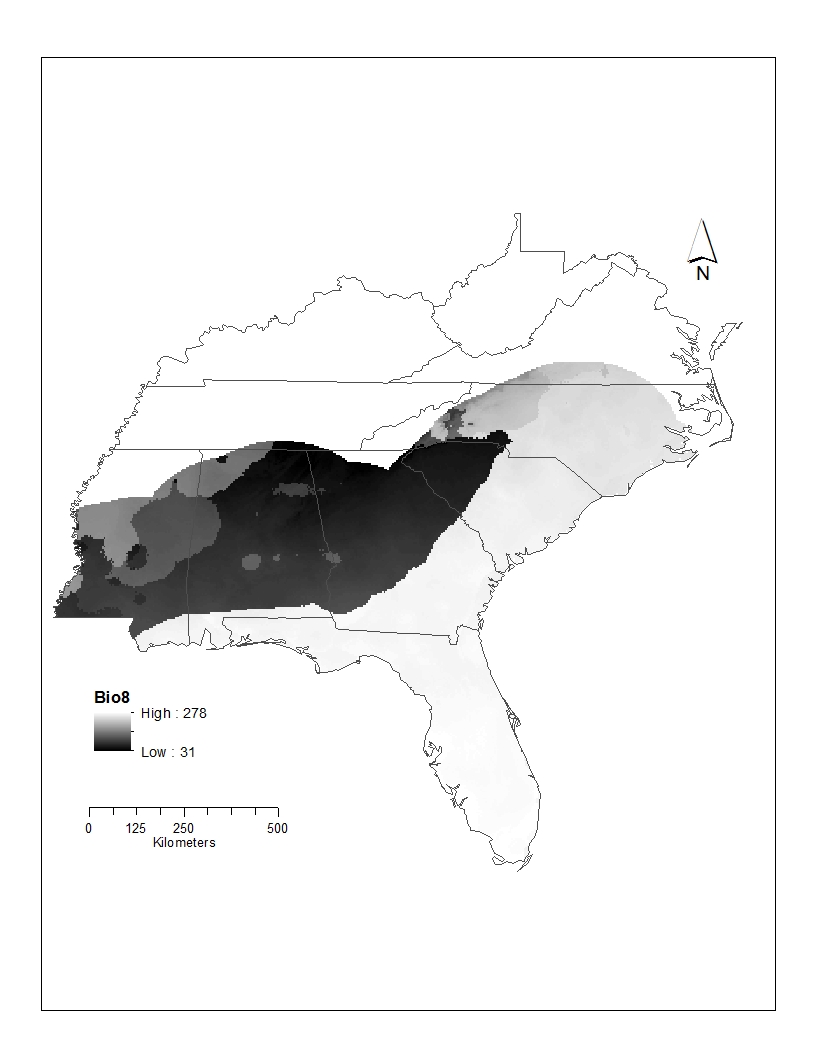

Supplement: Appendix S2 — Bioclimatic variables came from WorldClim ( http://www.worldclim.org/) and soil type from the Harmonized World Soil Database ( http://www.arcgis.com/home/item.html?id=1d16ed2a0aa24ab39e5ee6c491965883). Temperature is expressed in degrees Celsius multiplied by 10, precipitation is measured in mm, and soil type is a categorical variable with specific categories listed below. [file peerj-06-4647-s005.zip › Bio8.jpg]

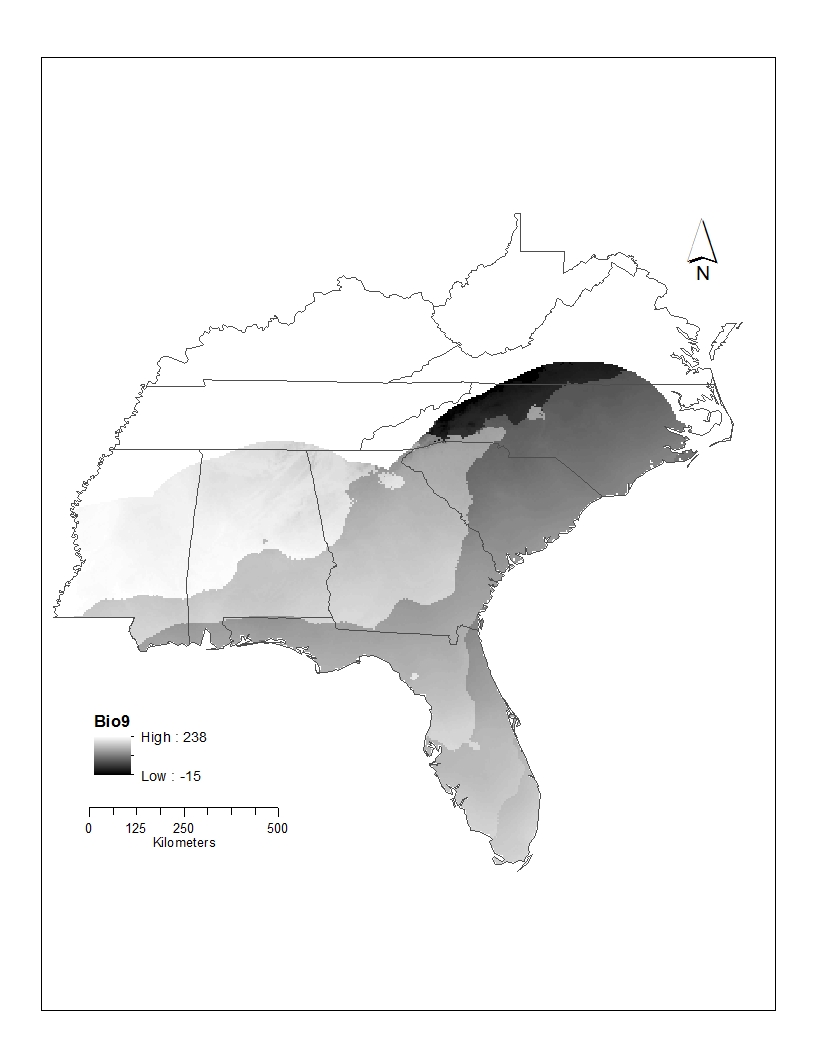

Supplement: Appendix S2 — Bioclimatic variables came from WorldClim ( http://www.worldclim.org/) and soil type from the Harmonized World Soil Database ( http://www.arcgis.com/home/item.html?id=1d16ed2a0aa24ab39e5ee6c491965883). Temperature is expressed in degrees Celsius multiplied by 10, precipitation is measured in mm, and soil type is a categorical variable with specific categories listed below. [file peerj-06-4647-s005.zip › Bio9.jpg]

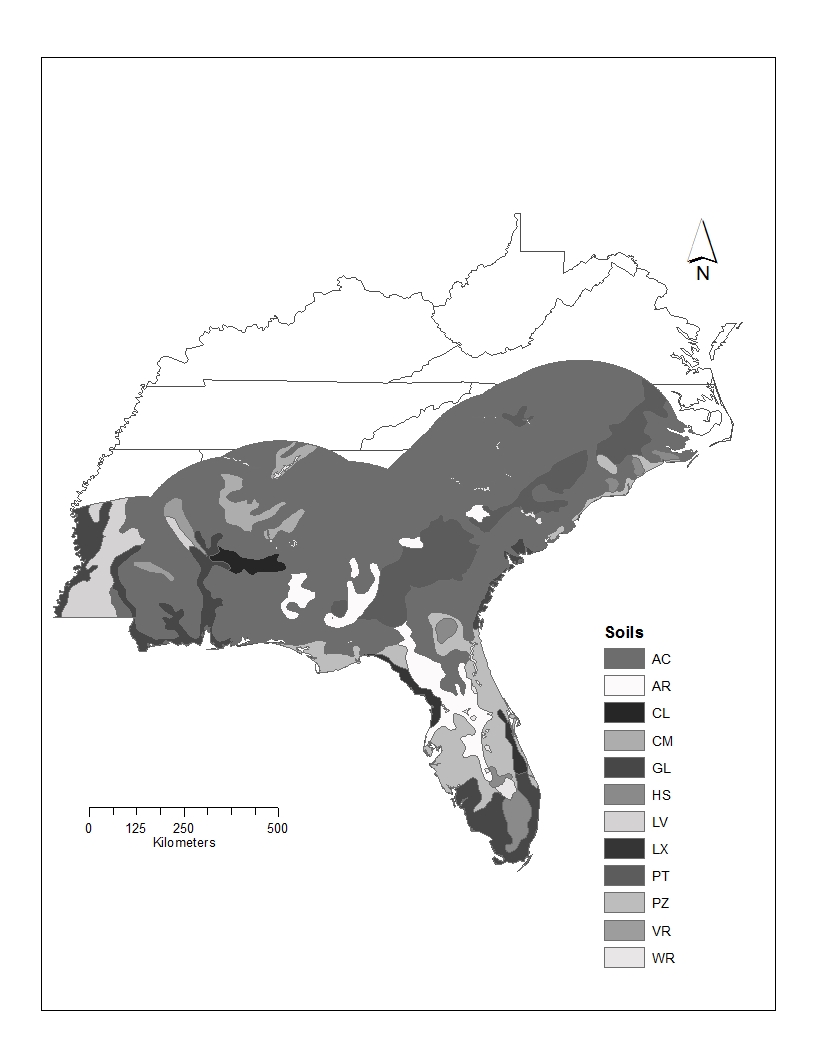

Supplement: Appendix S2 — Bioclimatic variables came from WorldClim ( http://www.worldclim.org/) and soil type from the Harmonized World Soil Database ( http://www.arcgis.com/home/item.html?id=1d16ed2a0aa24ab39e5ee6c491965883). Temperature is expressed in degrees Celsius multiplied by 10, precipitation is measured in mm, and soil type is a categorical variable with specific categories listed below. [file peerj-06-4647-s005.zip › Soil.jpg]

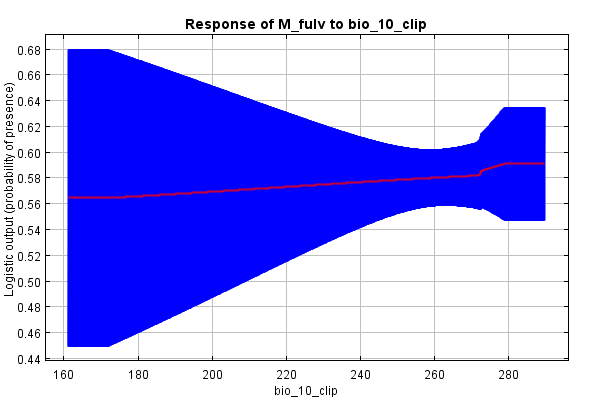

Supplement: Appendix S3 [file peerj-06-4647-s006.zip › Appendix 3/Bio10RC.png]

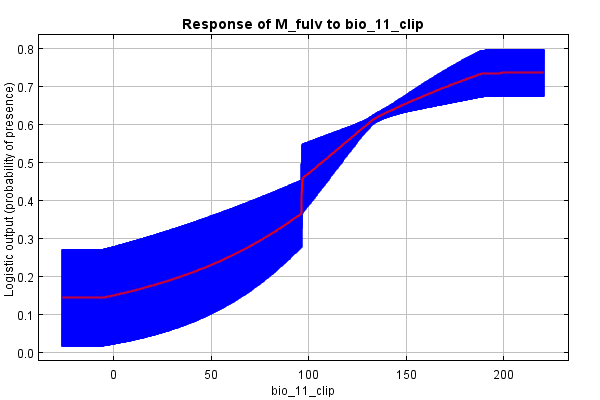

Supplement: Appendix S3 [file peerj-06-4647-s006.zip › Appendix 3/Bio11RC.png]

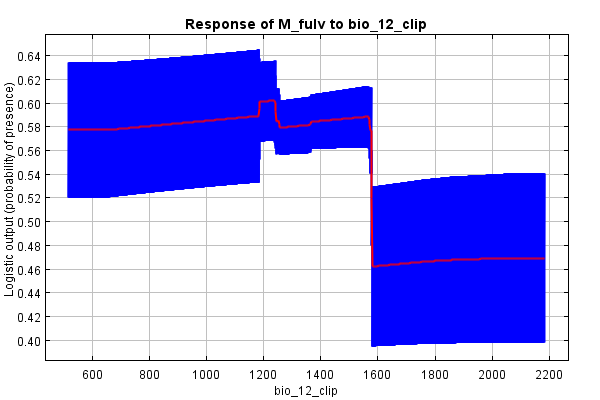

Supplement: Appendix S3 [file peerj-06-4647-s006.zip › Appendix 3/Bio12RCp.png]

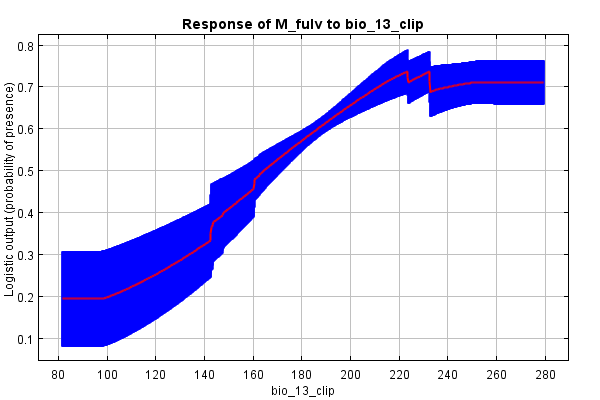

Supplement: Appendix S3 [file peerj-06-4647-s006.zip › Appendix 3/Bio13RC.png]

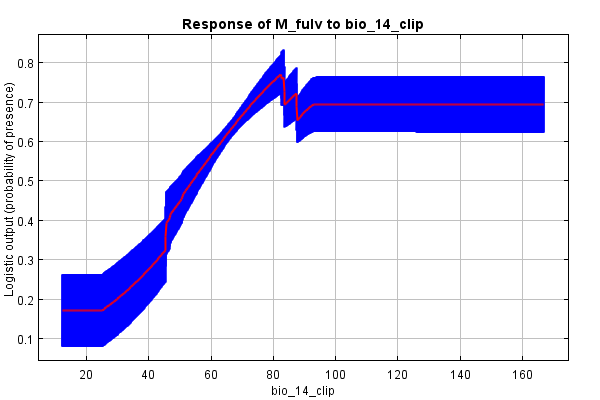

Supplement: Appendix S3 [file peerj-06-4647-s006.zip › Appendix 3/Bio14RC.png]

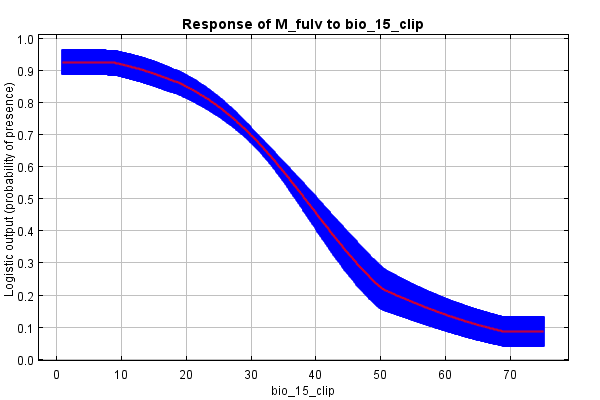

Supplement: Appendix S3 [file peerj-06-4647-s006.zip › Appendix 3/Bio15RC.png]

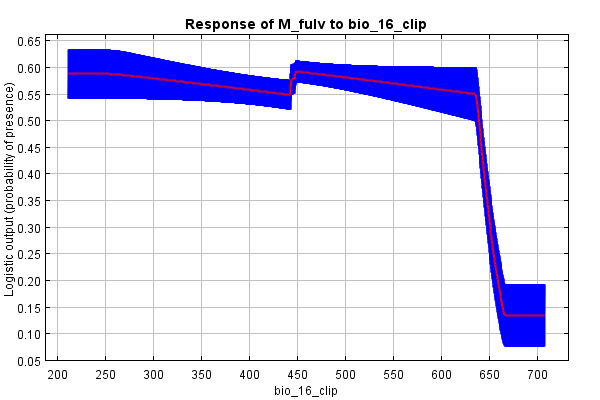

Supplement: Appendix S3 [file peerj-06-4647-s006.zip › Appendix 3/Bio16RC.png]

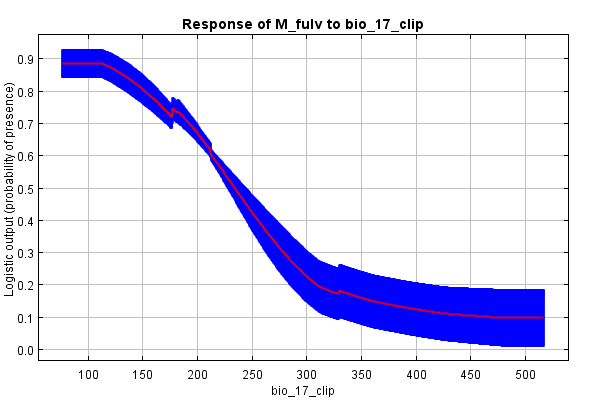

Supplement: Appendix S3 [file peerj-06-4647-s006.zip › Appendix 3/Bio17RC.png]

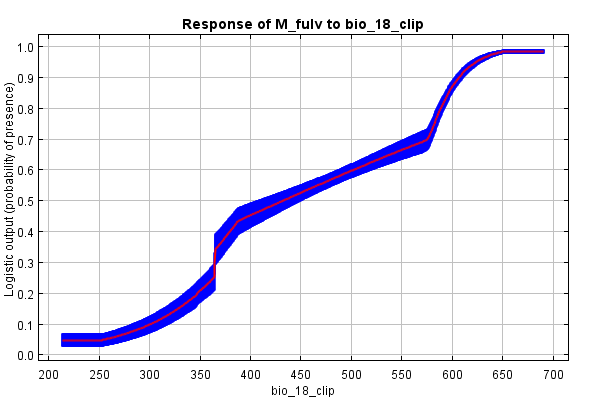

Supplement: Appendix S3 [file peerj-06-4647-s006.zip › Appendix 3/Bio18RC.png]

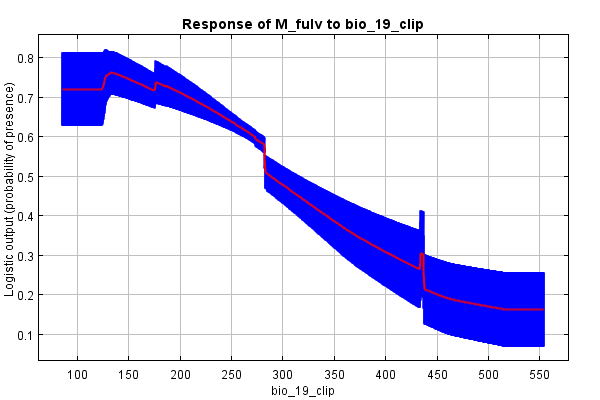

Supplement: Appendix S3 [file peerj-06-4647-s006.zip › Appendix 3/Bio19RC.png]

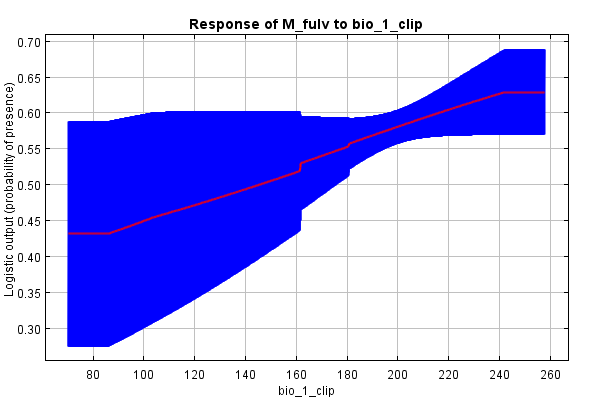

Supplement: Appendix S3 [file peerj-06-4647-s006.zip › Appendix 3/Bio1RC.png]

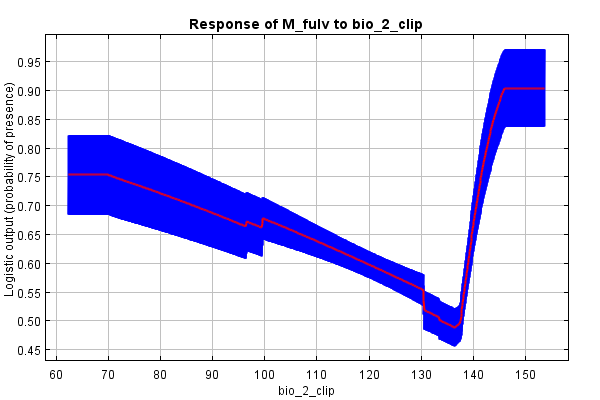

Supplement: Appendix S3 [file peerj-06-4647-s006.zip › Appendix 3/Bio2RC.png]

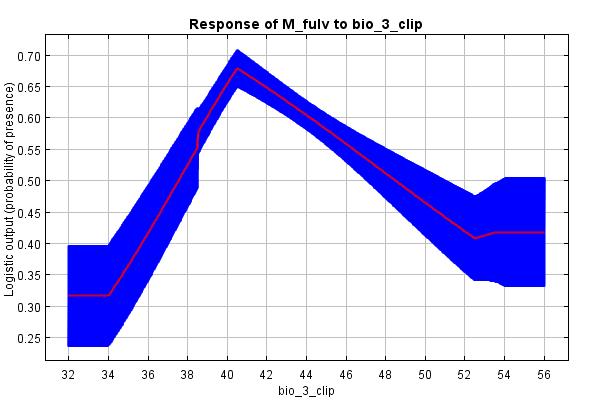

Supplement: Appendix S3 [file peerj-06-4647-s006.zip › Appendix 3/Bio3RC.png]

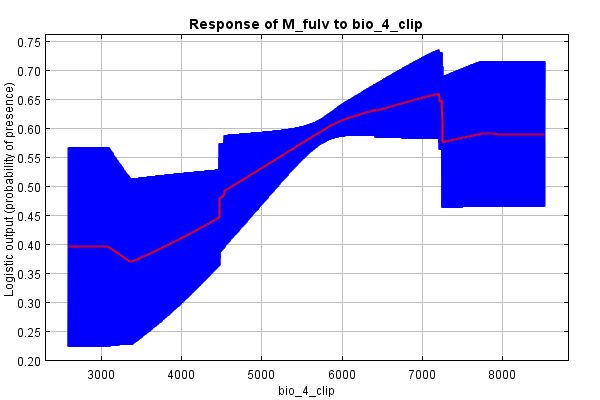

Supplement: Appendix S3 [file peerj-06-4647-s006.zip › Appendix 3/Bio4RC.png]

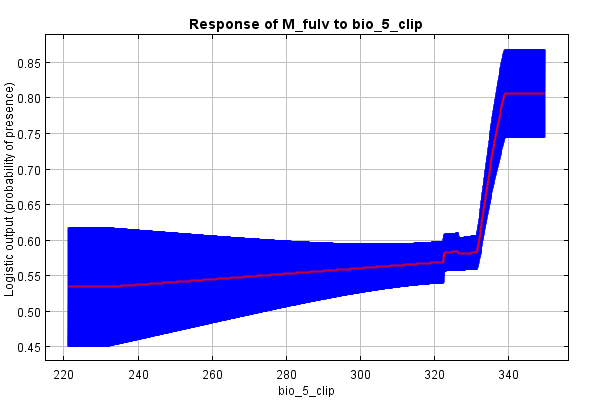

Supplement: Appendix S3 [file peerj-06-4647-s006.zip › Appendix 3/Bio5RC.png]

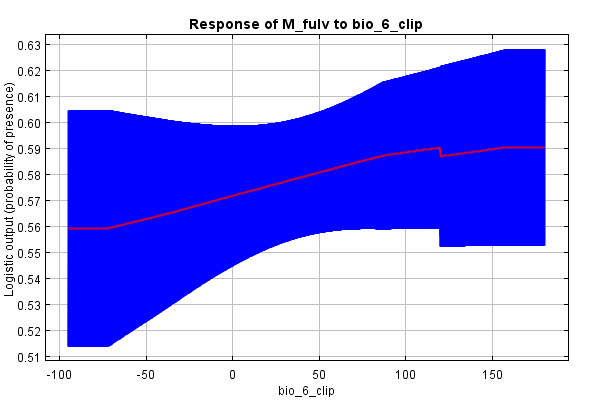

Supplement: Appendix S3 [file peerj-06-4647-s006.zip › Appendix 3/Bio6RC.png]

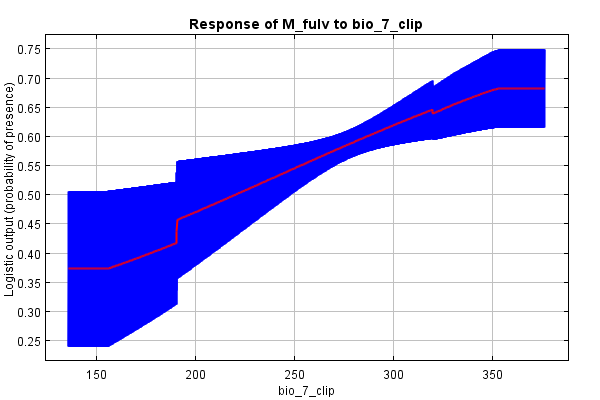

Supplement: Appendix S3 [file peerj-06-4647-s006.zip › Appendix 3/Bio7RC.png]

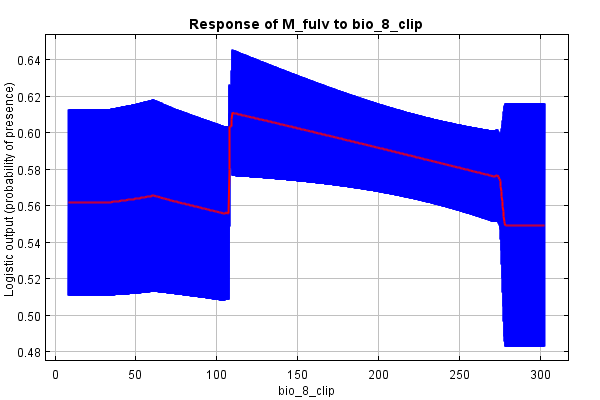

Supplement: Appendix S3 [file peerj-06-4647-s006.zip › Appendix 3/Bio8RC.png]

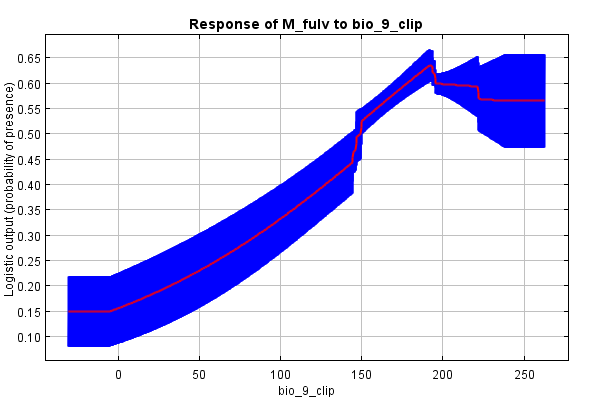

Supplement: Appendix S3 [file peerj-06-4647-s006.zip › Appendix 3/Bio9RC.png]

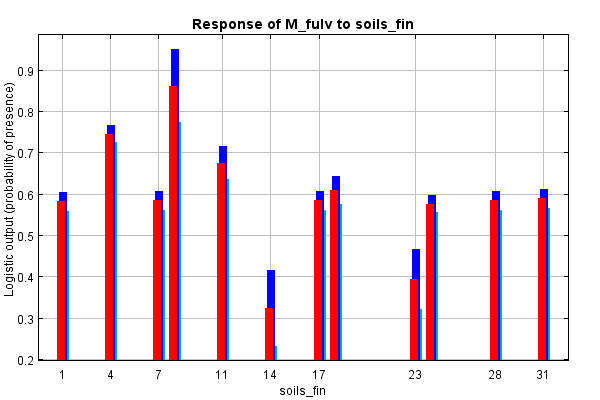

Supplement: Appendix S3 [file peerj-06-4647-s006.zip › Appendix 3/SoilRC.png]
