# Supplementary material for: Is the future already here? The impact of climate change on the distribution of the eastern coral snake (Micrurus fulvius)
Source: PeerJ. 2018 May 1;6:e4647. doi: 10.7717/peerj.4647 (PMC5935076; doi:10.7717/peerj.4647)
Supplement: Appendix S3 [file peerj-06-4647-s006.zip › Appendix 3/Appendix S3.docx]

Appendix S3. Response curves from each environmental layer used in the current climate modeling for the Full model.

Bio1RC = Annual Mean Temperature
Bio2RC = Mean Diurnal Range (Mean of monthly (max temp - min temp))
Bio3RC = Isothermality (BIO2/BIO7) (* 100)
Bio4RC = Temperature Seasonality (standard deviation *100)
Bio5RC = Max Temperature of Warmest Month
Bio6RC = Min Temperature of Coldest Month
Bio7RC = Temperature Annual Range (BIO5-BIO6)
Bio8RC = Mean Temperature of Wettest Quarter
Bio9RC = Mean Temperature of Driest Quarter
Bio10RC = Mean Temperature of Warmest Quarter
Bio11RC = Mean Temperature of Coldest Quarter
Bio12RC = Annual Precipitation
Bio13RC = Precipitation of Wettest Month
Bio14RC = Precipitation of Driest Month
Bio15RC = Precipitation Seasonality (Coefficient of Variation)
Bio16RC = Precipitation of Wettest Quarter
Bio17RC = Precipitation of Driest Quarter
Bio18RC = Precipitation of Warmest Quarter
Bio19RC = Precipitation of Coldest Quarter

SoilRC = Soil Type, codes are listed below:

AC = Soils with subsurface accumulation of low activity clays and low base saturation

AR = Sandy soils featuring very weak or no soil development

CL = Soils with accumulation of secondary calcium carbonates

CM = Weakly to moderately developed soils

GL = Soils with permanent or temporary wetness near the surface

HS = Soils which are composed of organic materials

LV = Soils with subsurface accumulation of high activity clays and high base saturation

LX = Soils with subsurface accumulation of low activity clays and high base saturation

PT = Wet soils with an irreversibly hardening mixture of iron, clay and quartz in the

Subsoil

PZ = Acid soils with a subsurface accumulation of iron-aluminum-organic

VR = Dark-colored cracking and swelling clays

WR = Water Bodies
